# The Visibility of Changes in the Antioxidant Compound Profiles of Strawberry and Raspberry Fruits Subjected to Different Storage Conditions Using ATR-FTIR and Chemometrics

**DOI:** 10.3390/antiox12091719

**Published:** 2023-09-05

**Authors:** Monika Sachadyn-Król, Iwona Budziak-Wieczorek, Izabella Jackowska

**Affiliations:** Department of Chemistry, Faculty of Food Sciences and Biotechnology, University of Life Sciences in Lublin, Akademicka 15, 20-950 Lublin, Poland; monika.sachadyn-krol@up.lublin.pl (M.S.-K.); izabella.jackowska@up.lublin.pl (I.J.)

**Keywords:** FTIR spectroscopy, antioxidants, phenolics, berries, ozone, chemometrics

## Abstract

Strawberry cultivars Portola and Enduro, as well as raspberry cultivars Enrosadira and Kwazi, were evaluated for their antioxidant potential after treatment with gaseous ozone and different refrigeration storage conditions. Their antioxidant capacity was investigated with ABTS and DPPH methods, and the chemical composition was determined by measuring the total phenolic (TPC) and flavonoid (TFC) compounds. The classification of different samples of berry puree was influenced significantly by both the cultivars and the refrigeration storage method. Moreover, FTIR spectroscopy coupled with chemometrics was used as an alternative technique to conventional methods to determine the chemical composition of strawberries and raspberries. The chemometric discrimination of samples was achieved using principal component analysis (PCA), hierarchical clustering analysis (HCA) and linear discriminant analysis (LDA) modelling procedures performed on the FTIR preprocessed spectral data for the fingerprint region (1800–500 cm^−1^). The fingerprint range between 1500 and 500 cm^−1^, corresponding to deformation vibrations from polysaccharides, pectin and organic acid content, had a significant impact on the grouping of samples. The results obtained by PCA-LDA scores revealed a clear separation between four classes of samples and demonstrated a high overall classification rate of 97.5% in differentiating between the raspberry and strawberry cultivars.

## 1. Introduction

Vitamins A, C, and E, as well as certain phenolic compounds, carotenoids, and flavonoids present in fruits, exhibit anticancer properties and function as antioxidants. Antioxidants are effective scavengers of free radicals and also stimulate the production of detoxifying enzymes, which helps to eliminate toxic substances and mutagens from the body [1,2]. There is an increasing body of research confirming the relationship between low antioxidant intake and an increased risk of diseases such as cancer, cardiovascular diseases and many others [3]. Studies investigating the modification of plants with high levels of bioactive compounds have demonstrated that subjecting these plants to controlled stressors triggers a defense response. Under suitable conditions, this response results in an enhanced production of small-molecule antioxidants and the activation of specific enzyme groups. Abiotic elicitors like ozone are among the stressors that can induce changes in the chemical composition of plant material [4,5].

The use of ozone as a disinfectant is now common in various aspects of product storage, such as in storing products in an ozone-rich atmosphere, washing raw materials with ozonated water, or directly adding ozone to liquid products [6]. Ozone treatment is increasingly being used as an alternative and modern method to induce stress and enhance the health-promoting potential of plant materials. Despite numerous reports on ozone’s ability to induce an elicitation effect, this issue remains unclear. It appears that the effect of ozone treatment on the chemical composition of fruits and vegetables is influenced by several factors, including the type and variety of the product, the method and form of ozone treatment, and the dosage of ozone. Higher dosages and longer contact times can lead to increased oxidative stress, which negatively affects the quality of the product. Biologically active compounds can be oxidized through direct or indirect reactions [7]. Most reviewed articles indicate a decrease in vitamin C content and a deterioration of color, potentially due to chlorophyll degradation and browning reactions. Soft berries tend to exhibit an increase in anthocyanin content and higher susceptibility to ozone treatment [8].

Traditional approaches for assessing quality attributes and nutritional properties commonly rely on colorimetric or chromatographic techniques, such as LC-MS or HPLC [9]. However, these methods have several limitations. They are time-consuming, necessitate sample preparation and the use of chemical reagents, and are both labor-intensive and costly. Over the past few years, there has been growing interest in utilizing multivariate statistical techniques and advanced instrumentation to facilitate the use of rapid methods in predicting the concentration of specific chemical constituents [10]. FTIR applications combined with chemometric methods can provide alternative techniques to conventional methods to determine the quality traits of fruits or vegetables. The extent to which ozone modifies substances is still a subject of debate. Therefore, studying the stability and availability of biomolecules during digestion and fermentation processes is the initial step in gaining a better understanding of their bioaccessibility. Infrared techniques provide valuable, quick, and non-destructive means of examining changes in important molecules and structures within unaltered food samples [11]. Recently, many studies have paid particular attention to the possibility of measuring the quality, chemical composition and authentication of various food products, using FTIR spectroscopy coupled with chemometric analysis [12,13,14,15,16]. The interest in this method is essentially due to the rapidity of the method, the possibility of determining several quality traits simultaneously and the ease of implementation. FTIR is employed to assess various frequently studied chemometric processes involving plant secondary metabolites [17]. For example, Falcioni et al. [18] indicated that every variety of lettuce possesses distinctive spectral patterns of secondary metabolites when analyzed through ATR-FTIR spectroscopy. These processes rely on vibrational groups and distinct band properties, and the patterns, visually represented through a heatmap, hold promise as a valuable tool for grouping varieties. Infrared spectroscopy profiles unveiled distinct molecular structural variations between lignin and other carbohydrates within oats and barley. FTIR analysis successfully discerned functional groups specific to carbohydrates and enabled the prediction of nutritional composition within grains, as demonstrated by Prates et al. [19]. ATR-FTIR spectroscopy proved to be a rapid and non-invasive technique for comprehensive analysis of the chemical composition of sorghum flour, distinguishing tannins, identifying variations in the protein secondary structure within endosperm fractions, and estimating protein content even with limited sample sizes [20]. Interestingly, the FTIR protocol was devised for the direct quantification of the active constituents—carvacrol, thymol, and p-cymene—within oregano and thyme essential oils [21]. The authors concluded that the developed FTIR approach exhibited swifter results and a more economical profile compared to the conventional GC-MS technique.

Therefore, the objective of this research was to apply FTIR spectroscopy, coupled with the appropriate chemometric methods, to investigate the effect of the variety on the antioxidants compounds profile of strawberry and raspberry fruits and, the effect of ozonation and refrigeration storage conditions on the main bioactive compounds implied in the antioxidant capacity.

## 2. Materials and Methods

### 2.1. Plant Material and Extract Preparation

Healthy, fully ripe and appropriately shaped strawberry fruits (*Fragaria* × *ananassa Duchesne*) of two varieties (S1—Portola and S2—Enduro) and raspberries (*Rubus idaeus*) of two varieties (R1—Enrosadira and R2—Kwazi) ready for immediate consumption were used for the research. The research material was obtained after cultivation in Zienki (Lubelskie Province, Poland). Fruits were divided into groups of about 100 g. Two groups of fruit were the control samples, while the remainder were exposed to a stream of ozone gas capacity of 500 mg/h and pump capacity of 9 dm^3^/min at a temperature of 25 °C for 15, 30, 45 and 60 min. The N202C ozone generator was used. One batch of fruits was frozen immediately after ozonation, and the rest were stored for 24 h under refrigeration conditions and then also frozen. The fruits were stored in the dark at −18 °C in tightly sealed packages of LDPE film for 90 days. The control fruits (without ozone treatment) were also stored in similar conditions. The schematic diagram of the experiment is included in the Supplementary Materials (Scheme S1).

After thawing, the material was homogenized using POLYTRON^®^ PT 2500 E (Ecoline, Kinematica AG, Malters, Switzerland), then 5 g of each sample was weighed into the conical flasks, and 40 mL of 90% methanol (MeOH) (solution with 0.1% formic acid) was added. Then, an ultrasonic bath (10 min) was used and the material was left for 20 min. After this time, the sample was filtered into 50 mL volumetric flasks and made up to the mark with 90% methanol. The resulting methanol extract was used for further research.

### 2.2. Total Phenolic Compounds (TPC)

The phenolic compounds content in methanol extracts obtained from strawberry fruit was tested by the method with the Folin–Ciocalteu reagent [22]. Briefly, 120 µL of the extract was transferred to plastic cuvettes, then 1.56 mL of distilled water and 120 µL of the F-C reagent were added. This reagent was diluted 1:10 with distilled water immediately before the determination. Then, it was shaken and left for 6 min. After this time, 1.2 mL of a 7% Na_2_CO_3_ solution was added, and then the mixture was stored for 90 min in the dark at room temperature. The absorbance was measured at λ = 750 nm. The total content of phenolic compounds is given by conversion from the calibration curve as the gallic acid equivalent per 100 g of fresh fruit.

### 2.3. Total Flavonoid Content (TFC)

The flavonoid content in methanol extracts obtained from strawberry fruit was investigated using a spectrophotometric method based on the formation of a colored complex between flavonoids and aluminum chloride [23]. Briefly, 500 µL of extract was transferred to a test tube, and 3.2 mL of distilled water and 150 µL of 5% NaNO_2_ solution were added. Everything was mixed and left for 5 min. Then, 150 µL of 10% AlCl_3_ solution was added and it was left again for another 6 min. After this time, 1 mL of 1 M NaOH was added, mixed and the absorbance immediately measured at λ = 510 nm. The sum of flavonoids is expressed as the quercetin equivalent per 100 g fresh weight, based on a previously prepared standard curve for this compound.

### 2.4. Determination of Antioxidant Activity

The ABTS assay based on the determination of the degree of [2,2-azobis (3-ethylbenzothiazoline-6-sulfonate)] radical removal of van den Berg et al. (1999), slightly modified by Kim et al. (2003), was used here [22]. ABTS^+•^ was generated by reacting ABTS aqueous solution (7 mM) with potassium persulfate (2.45 mM) in the dark for 12–16 h. Stock solution was prepared by adding 1 mL of ABTS to 50 mL of ethanol, and then dosing it until the absorbance was 0.7 ± 0.02 at λ = 734 nm. The actual test was performed by collecting 3 mL of ABTS stock solution into a test tube, and then adding 20 µL of the tested extract. At the same time, a blank test was performed (the same amount of ethanol instead of the extract). Absorbance was measured at λ = 734 nm. The ABTS radical quenching capacity was expressed as the amount of Trolox equivalent per unit volume from the calibration curve.

The antioxidant activity was also determined using the DPPH (2,2-diphenyl-l-picryldrazyl) radical assay, based on the scavenging methods of Brand-Williams, Cuvelier, and Berset (1995) [24], slightly modified by Panich and Amatatongchai (2019) [25]. The stock solution was prepared by adding 50 mL of MeOH to 0.02 g DPPH. The solution was diluted again to the final concentration of the radical 0.04 mg/mL. The assay was performed by mixing 100 µL of the extract and 3 mL of DPPH solution and then storing in a dark place for 30 min. At the same time, a blank test was performed (the same amount of methanol instead of the extract). The absorbance at λ = 515 nm was then measured in a spectrophotometer. The quenching capacity of the DPPH radical was expressed as the amount of Trolox equivalent per unit volume from the calibration curve.

### 2.5. FTIR

FTIR spectra were measured using an IRSpirit FTIR spectrometer (Shimadzu, Kioto, Prefektura Kioto, Japan) equipped with a single reflection ATR accessory. Briefly, 5 μL of the fruit puree sample was spread on the surface of a ZnSe crystal with drying (N_2_ gas). Each spectrum was investigated at a range of 4500–500 cm^−1^ at 4 cm^−1^ intervals of spectral resolution by averaging 30 scans. For the multivariate FTIR, spectral data were pre-processed using the Grams/AI 8.0 software (Thermo Scientific, Waltham, MA, USA).

### 2.6. Statistical Analysis

In order to determine the significance of the impact that the respective factors had on the analyzed values, one and two-way variance analyses were performed. The significance of differences between the mean values was established using Student’s *t*-test and Turkey’s test. The adopted significance level was *p* < 0.05. A General Linear Model (GLM) with normal distribution and identity link functions was used to investigate the factors affecting the total phenolic content (TPC) and total flavonoids content (TFC) as well as the antioxidant activity against DPPH and ABTS for raspberry and strawberry fruits. The models included the plant phenolics level and antioxidant properties as the dependent variables and the cultivar, ozonation time and storage condition as fixed factors. The Wald test confirmed the existence of a significant statistically difference (*p* < 0.05). The statistical analysis was conducted using Statistica 13 software from StatSoft (TIBCO Software Inc., Palo Alto, CA, USA). All the tests and analyses were conducted in 3 replications.

Multivariate analyses, including principal component analysis (PCA), hierarchical cluster analysis (HCA) and linear discriminant analysis (LDA), were performed for the antioxidant activity and FTIR spectra. Grams/AI 8.0 software (Thermo Scientific, Waltham, MA, USA) was applied for multi-point baseline correction, Savitzky–Golay smoothing and Y offset correlation, and points were set to zero prior to the analysis. The PCA and HCA analyses were performed within the range of 1800–500 cm^−1^. In the hierarchical cluster analysis, the average linkage distances between the pairs of samples were used as linkage criteria. The Pearson correlation distance between the pairs of samples was assessed as a distance measure. Statistica 13 software (TIBCO Software Inc., Palo Alto, CA, USA) and OriginPro (OriginLab Corporation, Northampton, MA, USA) were applied for chemometrics analysis.

## 3. Results and Discussion

### 3.1. Phenolic Compounds Content and Antioxidant Activity

In this study, the effect of two cultivars of strawberry (S1—Portola and S2—Enduro) and raspberry (R1—Enrosadira and R2—Kwazi), the ozonation time and the storage method on the antioxidant activity and phenolic content in methanolic berry extracts were examined. First of all, one-dimensional results examining the influence of the selected factors on the total phenolic content (TPC), total flavonoids content (TFC) and antioxidant activity against DPPH and ABTS were presented. The results of the Student’s *t*-test that analyzed the influence of cultivars is presented in Table 1, and the effect of storage conditions in Table 2. Table S1 in the Supplementary Materials presents the averaged results obtained in this analysis, including the effects of the cultivar, storage conditions, and ozonation time.

In the analyzed raspberry samples, the variety primarily determined the content of phenolic compounds, flavonoids, and antioxidant activity (against DPPH and ABTS radicals) (Table 1). Among the analyzed raspberry fruits, the Kwazi showed a higher level of antioxidant activity and phenolic content. The Kwazi exhibited over twice the amount of flavonoids (106.37 mg quercetin/100 g FW) compared to the Enrosadira (50.27 mg quercetin/100 g FW). Fruits of variety R2 (Kwazi) demonstrated a significantly higher DPPH radical neutralization ability compared to variety R1 (Enrosadira), with a difference of up to 40%. On the other hand, for strawberry fruits, Portola showed a higher level of ABTS, TPC and TFC. The ability to quench the DPPH radical was higher (0.53 ± 0.04 mmol Trolox/100 FW) in the Enduro cultivar compared to Portola (0.45 ± 0.16 mmol Trolox/100 FW). The ABTS assay showed a higher antioxidant capacity, likely due to its ability to assess the antioxidant capacity of both hydrophilic and lipophilic compounds. In contrast, the DPPH exclusively measured the capacity of hydrophobic compounds. These findings imply that when applied to a variety of plant foods containing hydrophilic, lipophilic, and high-pigmented antioxidant compounds, the ABTS assay is superior to the DPPH assay [23]. The content of phenolic compounds is affected by the species and variety of the plant and the growing conditions, as well as, at a later stage, by the time of post-harvest storage of the fruit [26].

The secondary metabolite content in plants and their antioxidant activity also depend on the storage method (Table 2). Therefore, in order to compare the changes resulting from this process, samples directly frozen after ozonation (0 h), as well as after 24 h refrigeration storage before freezing (24 h) were tested. In the case of raspberry extracts, no significant changes in the content of flavonoids, total phenolic compounds and DPPH were observed. It was also noted that 24 h storage after ozonation affects the antiradical activity of raspberry fruit in two ways. This process increased the ability to neutralize the DPPH radical, while reducing the ability to quench the ABTS radical. Storing strawberry fruit for 24 h in refrigeration conditions before freezing resulted in a statistically significant increase in the content of flavonoids (65.97 ± 24.4. as mg quercetin equivalents). At the same time, a statistically significant decrease in the content of phenolic compounds was also noted. A varied effect on the antiradical activity was observed. After 24 h storage, there was an increase in the ability to capture the ABTS radical and a decrease in the ability to capture the DPPH radical. These changes are statistically significant. These results show a significant effect of the storage time of strawberry fruit on the content of the bioactive compounds and the antioxidant activity. The content of phenolic compounds and flavonoids in stored strawberries may be related to metabolic changes. When the fruit is picked, the activity of endogenous antioxidant enzymes increases, which accelerates the synthesis of antioxidants. This increases the content of phenolic compounds and flavonoids, and the anti-radical activity [27].

**Figure 1:**
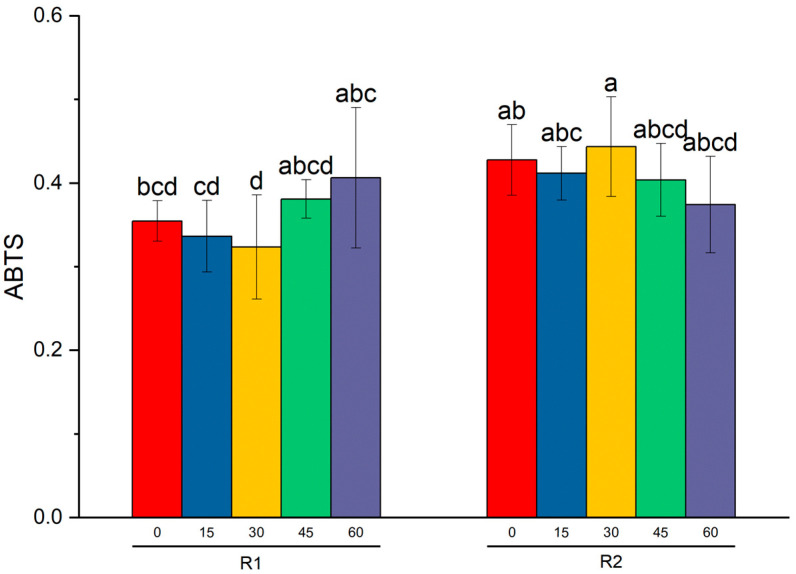
The results of Tukey’s test (significant level *p* < 0.05) and two-way ANOVA on the ABTS average level (± SD) for raspberries. Ozonation time and varieties were two factors used in tests. Different letters indicate significant differences between the means (*p* < 0.05).

Subsequently, the effect of ozonation time on the antioxidant properties and the content of bioactive compounds were studied by performing one-way and two-way ANOVA with Tukey’s test. The results for the Tukey’s test are presented in Table 3 and Figure S1 in the Supplementary Materials. For the two-way ANOVA (ozonation time × variations factors), results are presented in Table 4 and Figure 1. Based on the results of a one-factor analysis determining the impact of the ozonation process on the total flavonoid and total phenolic content in raspberry and strawberry fruits, slight variations were observed. However, these differences were not statistically significant. Moreover, changes in the antiradical activity in the method with ABTS and DPPH were also statistically insignificant for berries. Due to very large variations among the cultivars, two-way ANOVA was performed, applying the ozonation time and variety as factors. At the 0.05 level, the interactions between two factors were significant for ABTS for raspberries (Tukey’s test). The results show that the time of ozonation of the fruit slightly affected the content of bioactive compounds and the antioxidant activity. The raspberry variety studied significantly influenced the fruit’s response to ozone. These results are consistent with numerous other studies. In the group of different varieties and clones of Saskatoon berries, clones and varieties determined the levels of polyphenolic compounds in both ozonated and non-ozonated fruits [28]. The antioxidant activity measured by the DPPH and ABTS+ methods varied depending on the specific variety and clone being tested, as well as on the duration of ozone exposure. In other research, there were no significant variations observed in total soluble solids (TSS), titratable acidity (TA), total phenolic content (TPC), Trolox equivalent antioxidant capacity (TEAC), oxygen radical absorbance capacity (ORAC), or color characteristics between untreated and ozone-washed fruits (at both doses) immediately after treatment or during an 8-day storage period [29]. In general, the impact of ozone treatment is influenced by several key factors, including the type and variety of fruits or vegetables undergoing treatment, the specific form and method of ozone application, and the dosage of ozone used [8].

General linear models (GLM) were used to evaluate the hypothesis that three factors, cultivar, ozonation time and refrigerated storage condition, significantly affected the variation in flavonoid and phenolic content as well as antioxidant activity of raspberry and strawberry fruit samples. The Wald test confirmed the existence of significant statistical differences (*p* < 0.05), and the results of this test can be consulted in the Supplementary Material (Table S2 in Supplementary Material). In most cases, the obtained results were significantly influenced by the cultivars and storage condition, while the ozonation time was not important. In other words, the flavonoid and phenolic content and the antioxidant activity are significantly affected by the origin of the berries (cultivar) and the storage conditions in the refrigerator. Several research works have indicated that the content of phenolic compounds is associated with the botanical origins and post-harvest storage of the fruit [26,27]. On the other hand, when examining the effect in the intergroup system (cultivar × ozonation time × storage), no significant differences were found (*p* > 0.05).

### 3.2. Principal Components Analysis (PCA)

**Figure 2:**
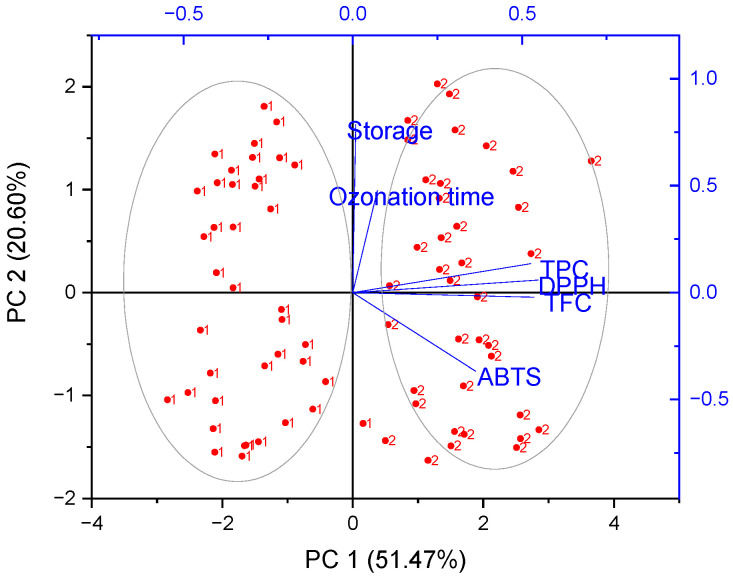
Biplot form of Principal Component Analysis (PCA) of the phenolic content (TFC, TPC), antioxidant capacities (DPPH and ABTS), ozonation time and storage conditions of two species of raspberry (1—Enrosadira and 2—Kwazi): 2D projection.

**Figure 3:**
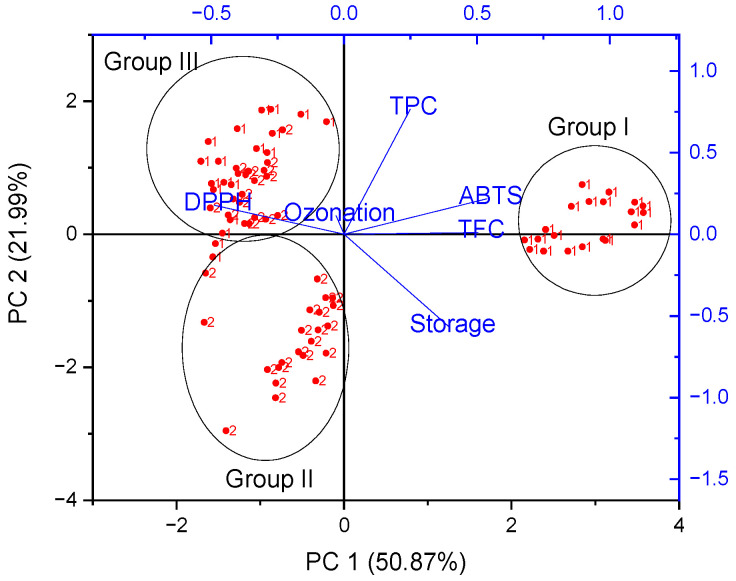
Biplot form of Principal Component Analysis (PCA) of the phenolic content (TFC, TPC), antioxidant capacities (DPPH and ABTS), ozonation time and storage condition of two species of strawberry (1—Portola and 2—Enduro): 2D projection.

Principal components analysis (PCA) was performed in order to obtain an overview of the similarities and differences between all the studied berry extracts [15,16]. Therefore, we investigated the relationship between the antioxidant activity, content of bioactive compounds, ozonation time and different storage methods (refrigerated storage) for two different cultivars of raspberries and strawberries. Fruit variety (1 and 2) was used as a grouping variable. The contributions of the principal components, eigenvalue and percentage of variance are shown in Table 5. A scree plot obtained from PCA is presented in Figure S2. The principal component analysis (PCA) showed that PC1 explained 51.47 and 50.87% of the total variances for raspberries and strawberries, respectively. The cumulative input from the first and second principal components reached 72.07% and 72.86% for raspberries and strawberries. Therefore, only the first two components (PC1 and PC2) were used in the further part of the analysis. Figure 2 and Figure 3 show a biplot that was used for the visualization of results from the PCA, as it combines both the principal component scores of the observations on the principal components and the loading vectors to represent the coefficients of the variables in a single 2D-dimensional plot [30]. PCA allowed the two different varieties of raspberries and strawberries to be allocated in separate parts in a biplot graph.

In the case of raspberry samples, it is possible to observe a strong positive correlation between TPC, TFC and DPPH (on the right), indicating that samples with the highest potency in scavenging the DPPH radical presented the highest TPC and TFC content (Figure 2). In addition, Enrosadira and Kwazi cultivar samples were on opposite sides of the PC1. The Kwazi cultivar was positively correlated with PC1, and showed higher antioxidant capacity and bioactive compounds (TPC and TFC) compared to the Enrosadira cultivar (Table 1). On the other hand, Enrosadira samples were negatively correlated with PC1.

Through the analysis of strawberries, score plots showed clustering effects, forming three separate groups (I–III). The first cluster was represented by selected samples of the Portola variety, the second cluster presents selected samples of the Enduro variety, while the third cluster forms a common group for both varieties. PC1 divided Portola samples (1) into two groups (I and III), which may be related to different storage methods. Moreover, PC2 also divided Enduro (2) samples into two groups (II and III). In the case of strawberries, not only did the variety affect the differentiation of samples, but also the storage condition. A strong positive correlation between TFC and ABTS can be seen (on the right), indicating that samples with the highest potency in scavenging the ABTS radical presented the highest TFC, namely group I. On the other hand, DPPH (on the left) showed a negative correlation to ABTS and TPC. The time of ozonation was in the middle of the graph and did not affect the division of strawberry samples.

### 3.3. FTIR Spectroscopy

FTIR spectroscopy is a simple, rapid and accurate method for simultaneously determining sugars, pectin and organic acid contents (citric acid) in natural fruit products like strawberries and raspberries [31,32]. Table 6 presents all the characteristic peak maxima and assigns the vibrations of the corresponding functional groups. Figures S3–S6 in the Supplementary Materials show FTIR spectra measured for samples of two cultivars of raspberries and strawberries that were normalized to a maximum around the 3220 cm^−1^ band. In the case of all studied samples, similar spectral properties were observed. However, when comparing the intensities of some peaks, clear differences between raspberry and strawberry samples were observed.

**Figure 4:**
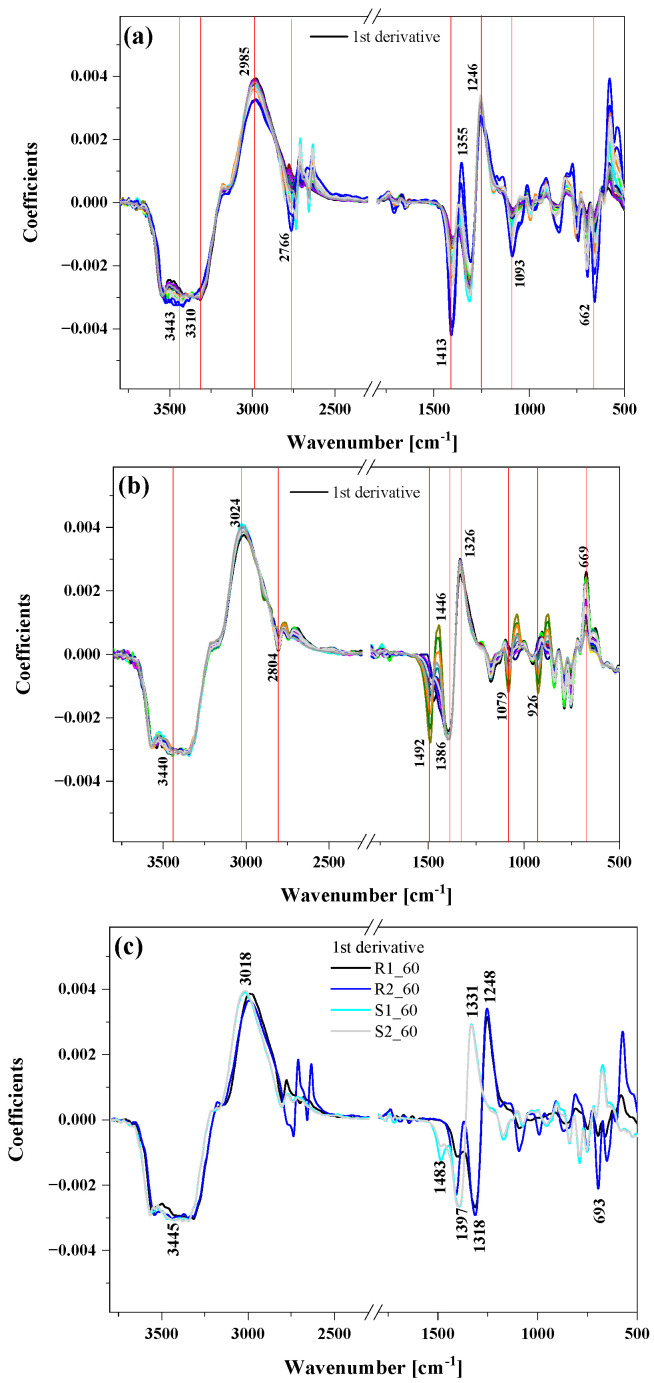
The pre-processed (smoothing with 20 windows + 1st derivative) FTIR spectra of all raspberries (panel (**a**)), all strawberries (panel (**b**)) and the comparison of two selected samples of raspberries (R1, R2) and strawberries (S1, S2) after 60 min of ozonation (panel (**c**)).

The FTIR spectra of the puree samples showed absorption bands at various frequencies that were related to the presence of various functional groups that are part of the chemical compounds present in food samples, such as alcohols, phenols, aldehydes, sugars and amino acids. To facilitate the analysis, discussion and comparison of the results obtained, the spectra were pre-processed using the first derivative (Figure 4). After the pre-processing, the FTIR spectra of all raspberry and strawberry purees with the most variability could be observed at around 3600–3100 cm^−1^, 3100–2800 cm^−1^ and 1500–1050 cm^−1^ (Figure 4a–c).

All the selected samples for this study had a very broad and strong band in the range of 3600–3000 cm^−1^, mainly resulting from stretching vibrations of hydroxyl groups, ν(-OH) present in water, phenolic compounds and organic acids (citric acid) [33]. Absorption bands at about 3024 and 2985 cm^−1^, as well as 2804 and 2985 cm^−1^, corresponded to asymmetric and symmetric stretching vibrations of CH_2_ and CH_3_ groups of aliphatic compounds [34]. Additionally, bands in the range of 3000–2800 cm^−1^ were most likely due to stretching vibrations of C-H bonds present in hydrocarbons, O-H bonds of carboxylic acids and asymmetric stretching vibrations of C-H bonds of methyl (-CH_3_) groups in free phenolic acids and catechins [35].

The region of vibrations between 1800 and 1000 cm^−1^ (fingerprint region) is characterized mainly by vibrational modes absorbing in this region, including *ν*C–OH, *ν*C=C, *ν*C–C, *δ*CH_3_ and *δ*CH_2_, the *δ*C–O–C of the glycosidic linkage and *δ*COH, and showed noticeable differences among groups of strawberries and raspberries. The wavenumber range from 1700 to 1500 cm^−1^ corresponded to the region of C-O stretching vibrations of pectin and acids, but these absorption bands were overlapped by the water absorption band [36]. The band at around 1730–1750 cm^−1^ indicates the presence of the esterified carbonyl group (C=O) observed in pectin. The region at 1540 cm^−1^ is due to the deformation movements of the hydroxyl group –OH.

Sugars (such as glucose and fructose) and organic acids (such as citric and malic acids) presented intense and characteristic bands in the region between 1500 and 950 cm^−1^ [37]. The region from 1500 to 1200 cm^−1^ showed a combination of bands of low intensity that could be related to –CH_2_, –OCH, –COH, and –CCH deformations characteristic of fructose [19]. Multiple bands can also be assigned to the C–O stretching absorption and C–O–H bending of phenols, carboxylic acids and carbohydrates. The fingerprint region, from 1200 to 900 cm^−1^, showed stretching vibrations of C–C and C–O bonds, also related to organic acids, disaccharides, polysaccharides, and glycosylated anthocyanins [12]. Finally, at 950–930 cm^−1^, pectin-characteristic balancing vibrations occurred.

Specifically, differences in the FTIR spectra indicated qualitative and quantitative differences in phenolic compounds, organic acids and carbohydrates in strawberry and raspberry fruits. Thus, an FTIR spectral analysis of different berry samples could be a useful tool for investigating levels of specific chemical compounds. The spectral analysis showed differences in the region for groups related to sugar (glucose and fructose). These differences were most likely related to the cultivars used. To confirm the differences between samples, multivariate analysis techniques such as PCA and HCA were performed.

### 3.4. Principal Component Analysis (PCA) and Hierarchical Cluster Analysis HCA for FTIR Spectra

To better understand FTIR spectra variability, principal component analysis (PCA) and hierarchical clustering analysis (HCA) were performed. In order to evaluate the main differences in the FTIR spectra of the berry purees, multivariate analysis was applied to the region of 1800–500 cm^−1^ by generating two-dimensional plots using two principal components, PC1 and PC2. Moreover, to facilitate the analysis, discussion, and comparison of the results obtained from FTIR, spectroscopy analyses were performed on normalized data as well as for the spectra that were normalized and pre-processed using the first derivative. PCA and HCA were performed separately for strawberries and raspberries with different ozonation times and storage methods showed no significant differences). This indicates the similarity in the FTIR spectra obtained for the tested samples. For that purpose, we decided to perform the multivariate analysis simultaneously for all raspberry and strawberry samples. In this case, it was determined that raspberries and strawberries have different spectral properties and can be easily distinguished from each other. The contribution of the principal components is shown in Table 7. Scree plots obtained from PCA are presented in Figures S7 and S8. The most common features among samples are generally expressed by the first few principal components (PCs). In our analysis, the first two principal components explained more than 85% (normalized data) and 94% (normalized data + 1st derivative) of the total variance (structure of dependence of primary variables) for the wavenumber range 1800–500 cm^−1^.

**Figure 5:**
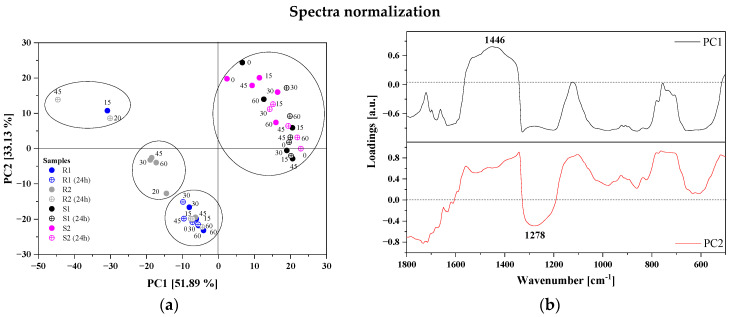

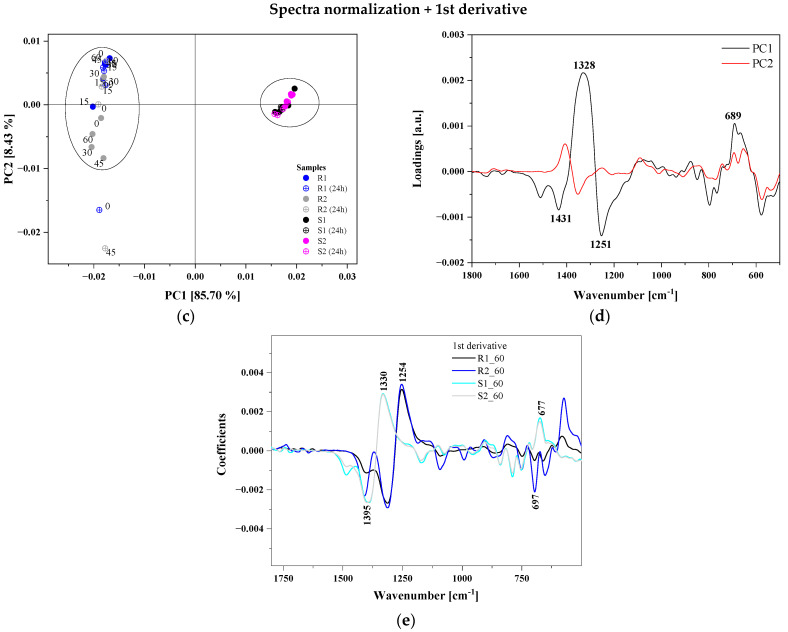
Results of PCA analysis: normalized data panel (**a**) PCA 2D score plots (PC1 vs. PC2), and panel (**b**) PC1 and PC2 correlation loading plots; the pre-processed spectra (normalization + 1st derivative) panel (**c**) PCA 2D score plots (PC1 vs. PC2), and panel (**d**) PC1 and PC2 correlation loading plots for the raspberry and strawberry samples (for the region 1800–500 cm^−1^). Comparison of two selected samples of raspberries (R1, R2) and strawberries (S1, S2) after 60 min of the ozonation—panel (**e**).

The PCA score plots for the first two PCs are shown in Figure 5a,c. In this case, for PC1 the contribution of the total variance for normalized data is 52%, while PC2 accounts for a lower percentage of the total variance (33%). On the other hand, pre-processing data with the first derivative significantly increased the participation of PC1 in explaining the variance of variables (85.7%). PCA performed on the fingerprint region revealed that the FTIR spectra were divided into two different groups. The PCA score plot of the first two PCs showed that strawberries were positively correlated with PC1, while raspberries were negatively correlated with PC2. Figure 5b,d present a loading plot that indicates which variables possessed the greatest influence on score, as well as which had the highest and the least contribution to creating the principal component. The PCA loading plot in Figure 5d reveals that PC1 was positively correlated with the bands at 1446, 1328 and 689 cm^−1^ assigned to deformation vibrations on -O-CH, C-OH and C-CH in the carbohydrate structure (fructose). The fingerprint region had significant influence on the classification between different samples of berries due to a unique spectrum specific for all polysaccharide, pectin and organic acid content.

Different ozonation times and storage methods did not cause significant differences in the FTIR spectra for selected berry cultivars.

**Figure 6:**
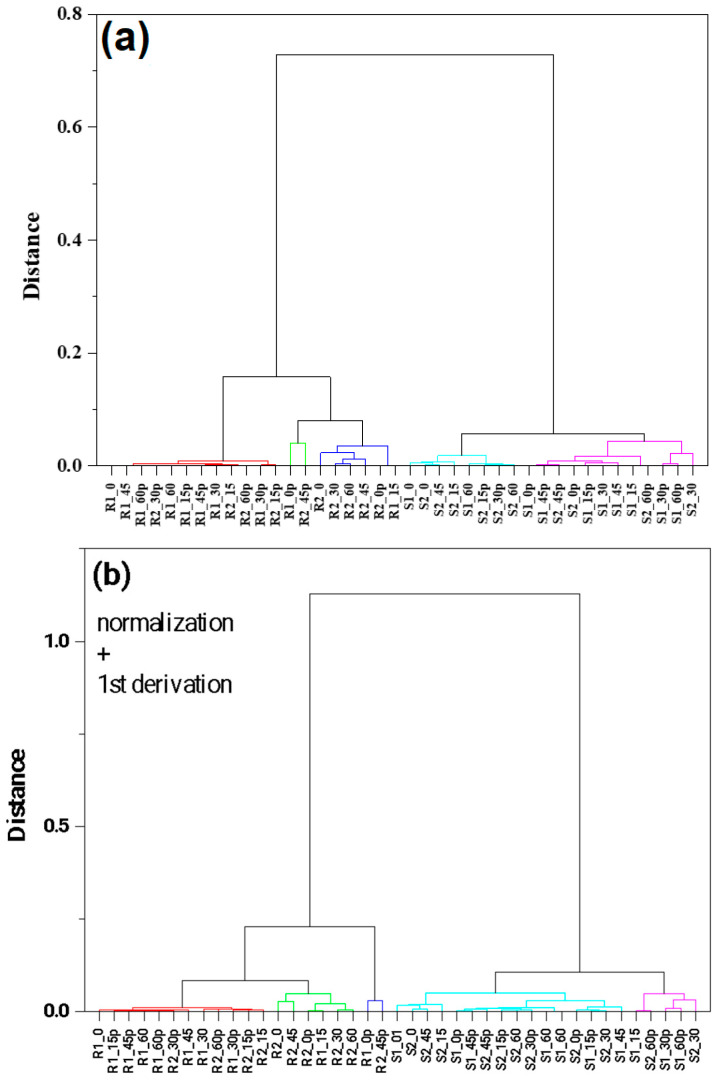
HCA analysis for the raspberry and strawberry samples for the region 1800–500 cm^−1^. Cluster method—group average. Clustering metric—Pearson correlation. Different colors indicate similarity cluster. Normalization at 3220 cm^−1^ (panel (**a**)), normalization + smoothing with 20 windows + 1st derivative (panel (**b**)).

Hierarchical clustering analysis was used to visualize the base classification of the group and the sub-group arrangement of the FTIR spectra of berry methanolic extracts. The HCA focused mainly on finding similarities between samples by the use of different classification algorithms. In this analysis, the same FTIR spectra, like in PCA, were taken into account. Figure 6a,b represents the three diagrams obtained from the hierarchical clustering analysis using the infrared spectra of the fingerprint region. The group average method linkage and Pearson correlation distance were used. The results were divided into five clusters in order to better visualize the data. In this case, similar results were obtained, like in PCA. Raspberry samples were divided into three groups, and strawberries into two. For the better classification of samples, supervised classification methods should be used. This is presented in the next paragraph.

### 3.5. PCA-LDA Classification Modelling

**Figure 7:**
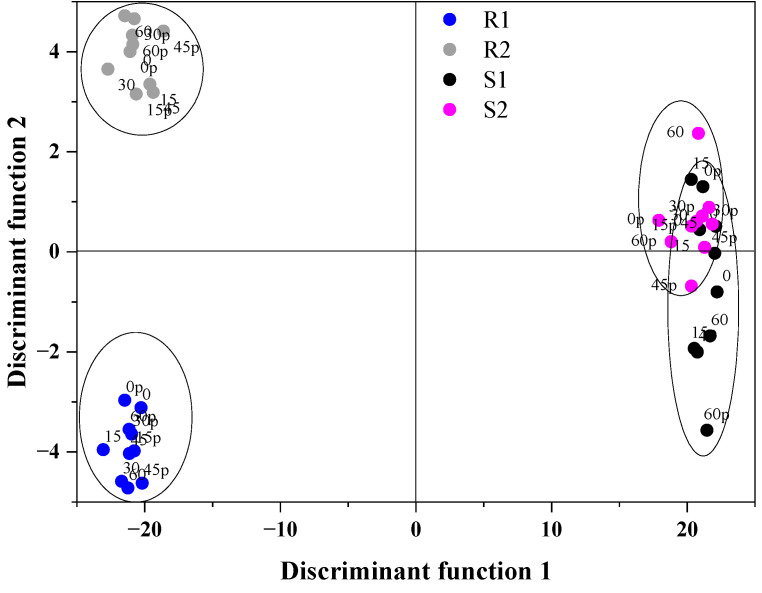
Linear discriminant score plot: R1—raspberries Enrosadira, R2—raspberries Kwazi, S1—strawberries Portola, S2—strawberries Enduro.

The classification of berry purees according to their cultivars (grouping variable) was performed for LDA based on the first three principal components (PC1, PC2 and PC3) and antioxidant properties (TFC, TPC, DPPH, ABTS) as dependent variables. For that purpose, PCA was applied on the infrared spectra in the fingerprint region (1800–500 cm^−1^) that was pre-processed with the first derivative (see Figure 4). The obtained results showed that all seven variables were statistically significant and should be used in the classification model. The samples were discriminated and the linear discriminant analysis resulted in two discriminant functions (LD) with the values of Wilks’s lambda of 6.895 × 10^−5^ and 0.034, respectively. Function 1 explained 97.81% of the total variance, while Function 2 explained 1.78%. The highest absolute value, which dominated the first discriminant function, was dominated positively by PC1, ABTS and DPPH, as well as negatively by TFC, TPC, PC2 and PC3. Figure 7 shows the LDA score plot representing the first two discriminant functions. The obtained PCA-LDA model was able to separate samples into four groups: R1, R2, S1 and S2. In fact, in the case of raspberries, a better division into groups was obtained; Enrosadira (R1) was on the negative side of LD2, while Kwazi (R2) was on the positive side of LD2. On the other hand, for strawberries, some samples could be classified into two groups. In Table 8, the classification matrix results of LDA are presented and 97.50% of the calibration set were correctly classified. The linear discriminant analysis was applied to all the physicochemical parameters, as well as spectral properties of well-defined berries, according to their cultivars.

## 4. Conclusions

This study presents the results of FTIR spectroscopy and antioxidant properties measured by traditional methods coupled with statistical analysis for two strawberry cultivars and two raspberry cultivars. The statistical analysis verified that the cultivars and different refrigeration storage conditions at a significance level of 0.05 had a significant impact on the phenolic and flavonoid content, as well as on the antioxidant properties of the fruit. However, it was shown that the ozonation time had no substantial impact on the differences in the obtained results for berry purees. In addition, FTIR spectroscopy coupled with multivariate analysis (PCA, HCA and LDA) was used as a fast method able to classify samples with great accuracy, as well as for an overview of the similarities and differences between the studied samples. PCA and PCA-LDA let us distinguish the types of cultivars among berry extracts and investigate the relationship between the antioxidant profile, ozonation time and different storage methods. The best discriminant quality was obtained when the first three principal components (PC1, PC2 and PC3) and antioxidant properties (DPPH, ABTS, TFC and TPC) were used simultaneously as variables. The result obtained using PCA-LDA scores equaled a clear separation between four classes of berries, according to the types of cultivars. Finally, the present findings reveal the potential of ATR-FTIR spectroscopy as a quick and easy-to-use technique to facilitate insights into spectral differences between various strawberry and raspberry varieties. Although there are numerous known limitations to FTIR, such as the relative intolerance of water (water interfere spectrum), its sensitivity to the physical properties of the analysis matrix and infrared light must change the dipole moment of a given bioactive compound.

## Figures and Tables

**Table 1:** The effect of the cultivar on the bioactive compound content and antioxidant activity.

	TPC ^1^	TFC ^2^	ABTS ^3^	DPPH ^3^
Raspberry				
Enrosadira	92.76 ± 10.77 ^a^	50.27 ± 3.17 ^a^	0.36 ± 0.06 ^a^	0.40 ± 0.04 ^a^
Kwazi	123.34 ± 10.36 ^b^	106.37 ± 8.53 ^b^	0.41 ± 0.05 ^b^	0.65 ± 0.06 ^b^
Strawberry				
Portola	174.22 ± 8.55 ^b^	58.51 ± 32.63 ^b^	0.91 ± 0.28 ^b^	0.45 ± 0.16 ^a^
Enduro	123.34 ± 10.36 ^a^	43.98 ± 9.25 ^a^	0.55 ± 0.07 ^a^	0.53 ± 0.04 ^b^

^1^ mg gallic acid/g fresh weight; ^2^ mg quercetin/100 g FW; ^3^ µmol Trolox/g fresh weight. The values are expressed as the mean ± SD (n = 3). Different letters in the same column for each fruit separately indicate significant differences between the means at *p* < 0.05 in the parametric Student’s *t*-test.

**Table 2:** The effect of storage conditions on the bioactive compounds content and antioxidant activity.

	TPC ^1^	TFC ^2^	ABTS ^3^	DPPH ^3^
Raspberry				
24 h	105.26 ± 17.75 ^a^	78.29 ± 30.03 ^a^	0.40 ± 0.06 ^b^	0.51 ± 0.13 ^a^
0 h	109.93 ± 19.30 ^a^	76.95 ± 28.20 ^a^	0.37 ± 0.05 ^a^	0.53 ± 0.14 ^a^
Strawberry				
24 h	167.87 ± 10.43 ^b^	36.52 ± 14.65 ^a^	0.60 ± 0.08 ^a^	0.57 ± 0.07 ^b^
0 h	160.73 ± 18.27 ^a^	65.97 ± 24.48 ^b^	0.86 ± 0.32 ^b^	0.41 ± 0.12 ^a^

^1^ mg gallic acid/g fresh weight; ^2^ mg quercetin/100 g FW; ^3^ µmol Trolox/g fresh weight. The values are expressed as the mean ± SD (n = 3). Different letters in the same column for each fruit separately indicate significant differences between the means at *p* < 0.05 in the parametric Student’s *t*-test.

**Table 3:** The results of Tukey’s test studying the effect of ozonation time on total phenolic content (TPC), total flavonoids content (TFC) and antioxidant properties (DPPH, ABTS) in extracts of raspberry and strawberry fruits.

**Raspberry**				
**Ozonation Time**	**TPC ^1^**	**TFC ^2^**	**ABTS ^3^**	**DPPH ^3^**
0	106.47 ± 18.06 ^a^	76.50 ± 29.73 ^a^	0.39 ± 0.05 ^a^	0.52 ± 0.13 ^a^
15	101.41 ± 20.02 ^a^	76.19 ± 27.73 ^a^	0.37 ± 0.05 ^a^	0.49 ± 0.15 ^a^
30	107.50 ± 21.26 ^a^	80.14 ± 31.93 ^a^	0.38 ± 0.09 ^a^	0.53 ± 0.17 ^a^
45	114.05 ± 16.56 ^a^	76.60 ± 28.64 ^a^	0.39 ± 0.04 ^a^	0.52 ± 0.14 ^a^
60	108.69 ± 16.84 ^a^	78.45 ± 30.14 ^a^	0.39 ± 0.07 ^a^	0.54 ± 0.12 ^a^
Mean	107.65 ± 18.63 ^a^	77.60 ± 28.92 ^a^	0.39 ± 0.06 ^a^	0.52 ± 0.14 ^a^
**Strawberry**				
**Ozonation time**	**TPC ^1^**	**TFC ^2^**	**ABTS ^3^**	**DPPH ^3^**
0	162.62 ± 13.65 ^a^	57.25 ± 32.43 ^a^	0.70 ± 0.30 ^a^	0.46 ± 0.09 ^a^
15	163.52 ± 19.68 ^a^	50.51 ± 21.73 ^a^	0.71 ± 0.31 ^a^	0.49 ± 0.16 ^a^
30	166.22 ± 10.01 ^a^	55.38 ± 23.62 ^a^	0.77 ± 0.23 ^a^	0.54 ± 0.12 ^a^
45	166.19 ± 18.49 ^a^	50.72 ± 28.75 ^a^	0.75 ± 0.27 ^a^	0.50 ± 0.09 ^a^
60	163.01 ± 13.77 ^a^	42.35 ± 14.90 ^a^	0.72 ± 0.25 ^a^	0.47 ± 0.14 ^a^
Mean	164.31 ± 15.21 ^a^	51.24 ± 24.93 ^a^	0.73 ± 0.27 ^a^	0.49 ± 0.12 ^a^

^1^ mg gallic acid/g fresh weight; ^2^ mg quercetin/100 g FW; ^3^ µmol Trolox/g fresh weight. The values are expressed as the mean ± SD (n = 3). Values in the same column not connected by the same letter are significantly different (*p* < 0.05).

**Table 4:** Tukey’s post hoc test (ozonation time vs. varieties) for raspberries.

Ozonation Time	Varieties	ABTS for Raspberries
0	R1	0.35 ± 0.02 ^bcd^
0	R2	0.43 ± 0.04 ^ab^
15	R1	0.34 ± 0.04 ^cd^
15	R2	0.41 ± 0.03 ^abc^
30	R1	0.32 ± 0.06 ^d^
30	R2	0.44 ± 0.06 ^a^
45	R1	0.38 ± 0.02 ^abcd^
45	R2	0.40 ± 0.04 ^abcd^
60	R1	0.41 ± 0.08 ^abc^
60	R2	0.37 ± 0.06 ^abcd^

Values in the same column not connected by the same letter are significantly different (*p* < 0.05).

**Table 5:** Eigenvalues, percentage of variance, and cumulative percentage in the data used for the PCA calculations obtained for the raspberries and strawberries.

Component	Principal Component Number	Eigenvalue	Percentage of Variance (%)	Cumulative (%)
Raspberries	1	3.08796	51.47%	51.47%
	2	1.23601	20.60%	72.07%
	3	0.94302	15.72%	87.78%
	4	0.51789	8.63%	96.41%
Strawberries	1	3.0524	50.87%	50.87%
	2	1.31927	21.99%	72.86%
	3	1.03032	17.17%	90.03%
	4	0.39886	6.65%	96.68%

**Table 6:** Location of the peaks of FTIR absorption bands, along with the assignment of relevant vibrations to the examined samples of raspberry (R1 and R2) and strawberry (S1 and S2) puree over the spectral range from 3800 to 500 cm^−1^.

Assignment	R1 (0 h)	R1 (24 h)	R2 (0 h)	R2 (24 h)	S1 (0 h)	S1 (24 h)	S2 (0 h)	S2 (24 h)
ν(–OH)	3225 3081	3223 3087	3225 3094	3220 3094	3223 3154	3228 3155	3240 3152	3242 3143
ν_as_(C–H)	2985	2985	2984	2984	3024	3024	3024	3024
ν_s_(C–H)	2791	2730	2747	2721	2804	2804	2792	2792
ν(C=O) ester carbonyl –COOR and carboxylate ion stretching –COO– in pectin	1741	1739	1742	1742	1745	1743	1748	1747
δ(–OH)	-	-	-	-	-		1536	1538
δ (–O–CH, C–OH, C–CH) from fructose	1370 1281	1368 1280	1382 1282	1385 1281	1460 1360	1456 1360	1453 1361	1452 1360
δ(C–O–C) from disaccharides, polysaccharides, and glycosylated anthocyanins	1030	1026	1029	1030	1037	1033	1031	1033
rocking (CH_3_) of pectin	950	945	948	945	930	933	935	934
δ(C–H)	826	822	827	828	829	829	826	830

**Table 7:** Eigenvalues, percentage of variance, and cumulative percentage in the data used for the PCA calculations obtained for the raspberry and strawberry samples from the FTIR spectra.

Component	Principal Component Number	Eigenvalue	Percentage of Variance (%)	Cumulative (%)
Raspberries and strawberries— spectra normalization	1	420.2715	51.88537	51.88537
	2	268.3365	33.12796	85.01333
	3	36.8148	4.54504	89.55837
	4	34.2225	4.22500	93.78337
Raspberries and strawberries— spectra normalization + 1st derivative	1	0.000331	85.69647	85.6965
	2	0.000033	8.42767	94.1241
	3	0.000011	2.84384	96.9680
	4	0.000006	1.56688	98.5349

**Table 8:** Classification matrix of the analyzed strawberries and raspberries using linear discriminant analysis (LDA) based on the phenolic content (TFC, TPC), antioxidant capacities (DPPH and ABTS) and the first three principal components.

From/To	R1	R2	S1	S2	% Correct
R1	10	0	0	0	100.00
R2	0	10	0	0	100.00
S1	0	0	9	1	90.00
S2	0	0	0	10	100.00
**Total**	**10**	**10**	**9**	**11**	**97.50**

## Data Availability

The datasets used and/or analyzed during the current study are available from the corresponding author upon reasonable request.

## Supplementary Materials

The following supporting information can be downloaded at: https://www.mdpi.com/article/10.3390/antiox12091719/s1, Scheme S1: Scheme of the plant material preparation experiment; Table S1: The results for the assays: antioxidant activity against DPPH and ABTS, the total phenolic content (TPC) and total flavonoids content (TFC) for two cultivars of raspberry (R1, R2) and strawberry fruits (S1, S2). Table S2: GLM results. The effects of cultivar, ozonation time, storage condition and interaction between studied parameters (ABTS, DPPH, TFC, TPC) for raspberry and strawberry fruits; Figure S1: Means plot and 95% confidence interval: for raspberries (panel a) and strawberries (panel b). Results of one-way analysis of variance (one-way ANOVA) taking into account the ozonation effect; Figure S2: Scree plot obtained from Principal Component Analysis (PCA) of the phenolic contents (TVC, TPC), antioxidant capacities (DPPH and ABTS), ozonation time and storage condition of raspberry (panel a) and strawberry (panel b); Figure S3: FTIR absorption spectra for the analyzed Enrosadira raspberry variety; Figure S4: FTIR absorption spectra for the analyzed Kwazi raspberry variety; Figure S5: FTIR absorption spectra for the analyzed Portola strawberry variety; Figure S6: FTIR absorption spectra for the analyzed Enduro strawberry variety; Figure S7: Scree plots (eigenvalues from principal components) for all examined samples of raspberry and strawberry from FTIR spectra (1800–500 cm^−1^ region); Figure S8: Scree plots (eigenvalues from principal components) for all examined samples of raspberry and strawberry from FTIR spectra (1800–500 cm^−1^ region). The pre-processed (smoothing with 20 windows + 1st derivation) FTIR spectra.
