# Supplementary material for: The Visibility of Changes in the Antioxidant Compound Profiles of Strawberry and Raspberry Fruits Subjected to Different Storage Conditions Using ATR-FTIR and Chemometrics
Source: Antioxidants (Basel). 2023 Sep 5;12(9):1719. doi: 10.3390/antiox12091719 (PMC10525253; doi:10.3390/antiox12091719)
Supplement: Supplementary file 1 [file antioxidants-12-01719-s001.zip › antioxidants-2508155-supplementary.pdf]

Supplementary materials

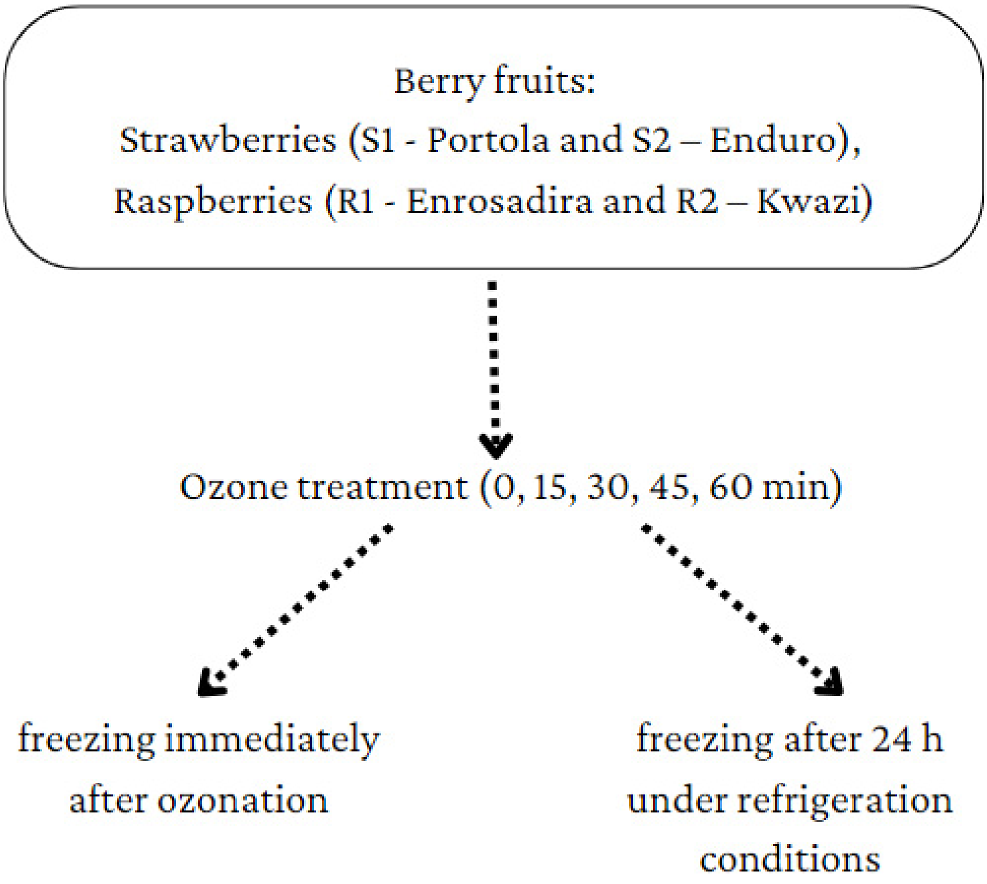

Scheme S1. Scheme of the plant material preparation experiment.

Table S1. The results for the assays: antioxidant activity against DPPH and ABTS, the total phenolic content (TPC) and total flavonoids content (TFC) for two cultivars of raspberry (R1, R2) and strawberry fruits (S1, S2). The results presented as mean values of triplicates.

Cultivar	Storage condition (h)	Ozonation time (min)	ABTS[1]	DPPH[1]	TFC[2]	TPC[3]
R1	0	0	0.3491	0.4217	52.0082	99.4557
R1	0	15	0.3639	0.4105	52.3515	88.3994
R1	0	30	0.3458	0.3412	50.2060	81.2795
R1	0	45	0.3208	0.3914	48.9186	86.1744
R1	0	60	0.3704	0.3499	50.2060	83.6072

R1	24	0	0.2954	0.3859	49.9485	90.7270
R1	24	15	0.3984	0.4191	50.6351	96.9569
R1	24	30	0.3704	0.3919	48.1462	102.2284
R1	24	45	0.4682	0.4342	50.5278	98.4545
R1	24	60	0.3445	0.4173	50.2060	95.6305
R2	0	0	0.4076	0.6321	110.0669	120.4046
R2	0	15	0.4675	0.6784	106.8486	123.2286
R2	0	30	0.4076	0.6132	106.2049	119.3777
R2	0	45	0.4158	0.6461	99.1246	120.0195
R2	0	60	0.4970	0.6945	119.7219	126.9083
R2	24	0	0.4117	0.6872	104.5314	126.9768
R2	24	15	0.4224	0.6421	102.3429	121.0464
R2	24	30	0.3854	0.6505	105.8831	134.6529
R2	24	45	0.3800	0.6254	108.5651	112.1894
R2	24	60	0.3710	0.6712	105.3038	125.2311
S1	0	0	0.5217	0.5226	27.3614	157.8070
S1	0	15	0.7015	0.6787	44.9775	179.7638
S1	0	30	0.6933	0.6828	44.2279	175.3983
S1	0	45	0.6880	0.5633	24.7377	179.1215
S1	0	60	0.6041	0.5226	19.8651	166.7950
S1	24	0	1.1952	0.3178	109.4453	181.6898
S1	24	15	1.1910	0.2626	83.5832	179.2500
S1	24	30	1.1499	0.3533	84.3328	171.8025
S1	24	45	1.1889	0.3470	95.9522	179.1215
S1	24	60	1.1356	0.2348	50.5997	171.4178
S2	0	0	0.5225	0.4983	40.8546	163.7133
S2	0	15	0.4782	0.5141	30.7347	152.2855
S2	0	30	0.6133	0.5495	37.1065	164.0985
S2	0	45	0.5690	0.5656	38.3535	169.2348
S2	0	60	0.6022	0.6062	56.9715	170.6473
S2	24	0	0.5525	0.5089	51.3494	147.2778
S2	24	15	0.4691	0.5127	42.7287	142.7835
S2	24	30	0.6108	0.5720	55.8471	153.5698
S2	24	45	0.5470	0.5124	43.8531	137.2623
S2	24	60	0.5525	0.5093	41.9790	143.1690

[1] μmol Trolox/g fresh weight, [2] mg quercetin/100 g FW, [3] mg gallic acid/g fresh weight. The values are expressed as the mean (n = 3).

Table S2. GLM results**.** The effects of cultivar, ozonation time, storage condition and interaction between studied parameters (ABTS, DPPH, TFC, TPC) for raspberry and strawberry fruits.

Raspberry				
			**ABTS**	
Parameter		df	Wald test	p
	Cultivar	**1**	**19.6816**	**0.0000***
	Ozonation time	4	1.8781	0.7582
	Storage	**1**	**8.0324**	**0.0046***
	*Cultivar*Ozonation time*Storage*	4	3.0637	0.5472
			**DPPH**	
Parameter		df	Wald test	p
	Cultivar	**1**	**609.3716**	**0.0000***
	Ozonation time	4	8.9061	0.0635
	Storage	1	1.8839	0.1699
	*Cultivar*Ozonation time*Storage*	4	2.6513	0.6178
			**TFC**	
Parameter		df	Wald test	p
	Cultivar	**1**	**1789.4363**	**0.0000***
	Ozonation time	4	7.1329	0.1290
	Storage	**1**	**4.6380**	**0.0313***
	*Cultivar*Ozonation time*Storage*	4	2.8590	0.5817
			**TPC**	
Parameter		df	Wald test	p
	Cultivar	**1**	**202.7267**	**0.0000***
	Ozonation time	**4**	**13.7308**	**0.0082***
	Storage	1	2.4686	0.1161
	*Cultivar*Ozonation time*Storage*	4	2.6265	0.6221
Strawberry				
			**ABTS**	
Parameter		df	Wald test	p
	Cultivar	**1**	**114.6871**	**0.0000***
	Ozonation time	4	2.2925	0.6821
	Storage	**1**	**61.3847**	**0.0000**
	*Cultivar*Ozonation time*Storage*	4	0.2959	0.9901
			**DPPH**	
Parameter		df	Wald test	p
	Cultivar	**1**	**22.7662**	**0.0000***
	Ozonation time	4	9.1366	0.0578
	Storage	**1**	**75.5393**	**0.0000***
	*Cultivar*Ozonation time*Storage*	4	3.2742	0.5130
			**TFC**	
Parameter		df	Wald test	p
	Cultivar	**1**	**13.3056**	**0.0003***
	Ozonation time	4	6.7077	0.1522

Storage	**1**	**54.6497**	**0.0000***
*Cultivar*Ozonation time*Storage*	4	3.4446	0.4864
		**TPC**	
Parameter	df	Wald test	p
Cultivar	**1**	**68.2383**	**0.0000***
Ozonation time	4	1.7138	0.7882
Storage	**1**	**8.8923**	**0.0029***
*Cultivar*Ozonation time*Storage*	4	4.2025	0.3793

*Values in the same column marked with an asterisk in superscript are statistically significant ($p < 0.05$).

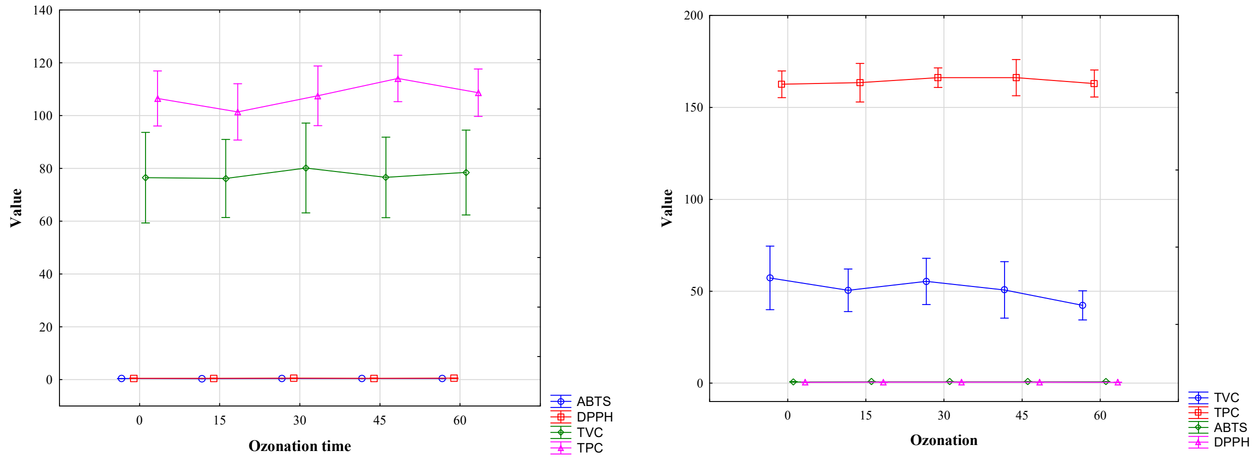

a) **b)**

Figure S1. Means plot and 95% confidence interval: for raspberries (panel a) and strawberries (panel b). Results of one-way analysis of variance (one-way ANOVA) taking into account the ozonation effect.

PCA– antioxidant properties, bioactive compound

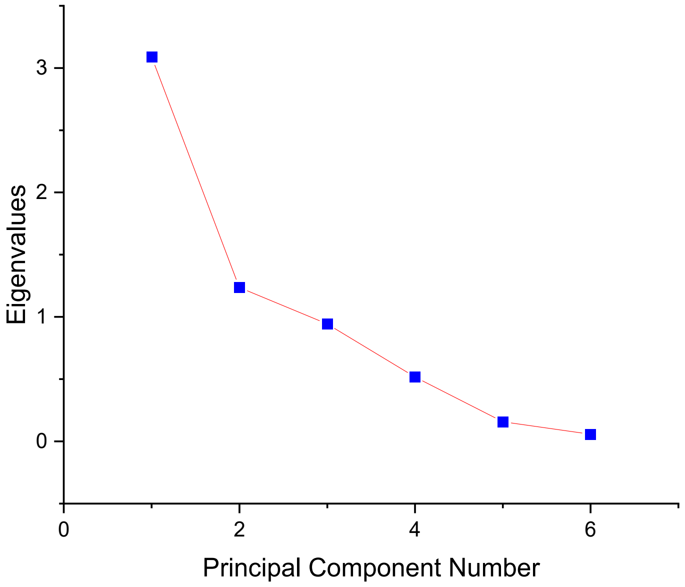

a)

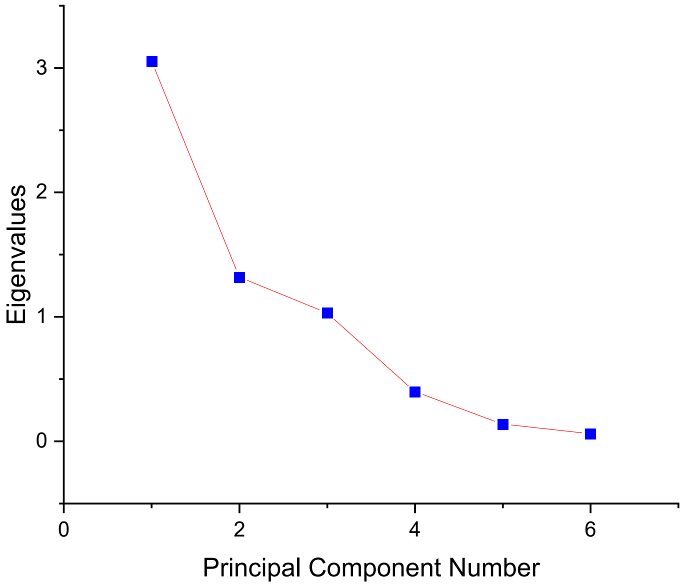

b)

Figure S2. Scree plot obtained from Principal Component Analysis (PCA) of the phenolic contents (TVC, TPC), antioxidant capacities (DPPH and ABTS), ozonation time and storage condition of raspberry (panel a) and strawberry (panel b).

FTIR spectroscopy

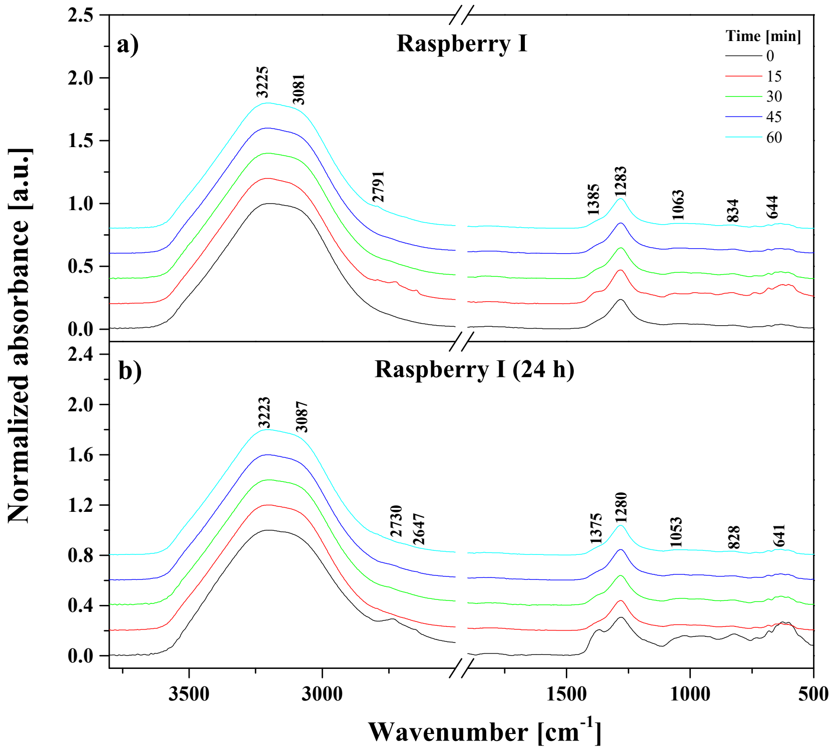

Figure S3. FTIR absorption spectra for the analyzed *Enrosadira* raspberry variety. One part of raspberry fruit was frozen immediately after ozonation (panel a). The second part of the raspberry fruit was stored for 24 h under refrigeration conditions and then also frozen (panel b).

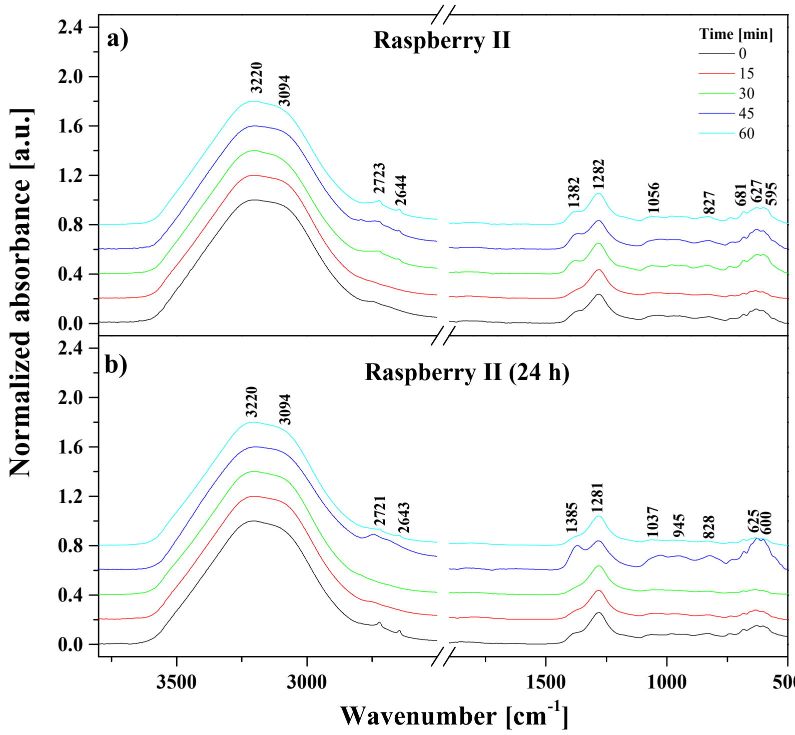

Figure S4. FTIR absorption spectra for the analyzed *Kwazi* raspberry variety. One part of raspberry fruit was frozen immediately after ozonation (panel a). The second part of the raspberry fruit was stored for 24 h under refrigeration conditions and then also frozen (panel b).

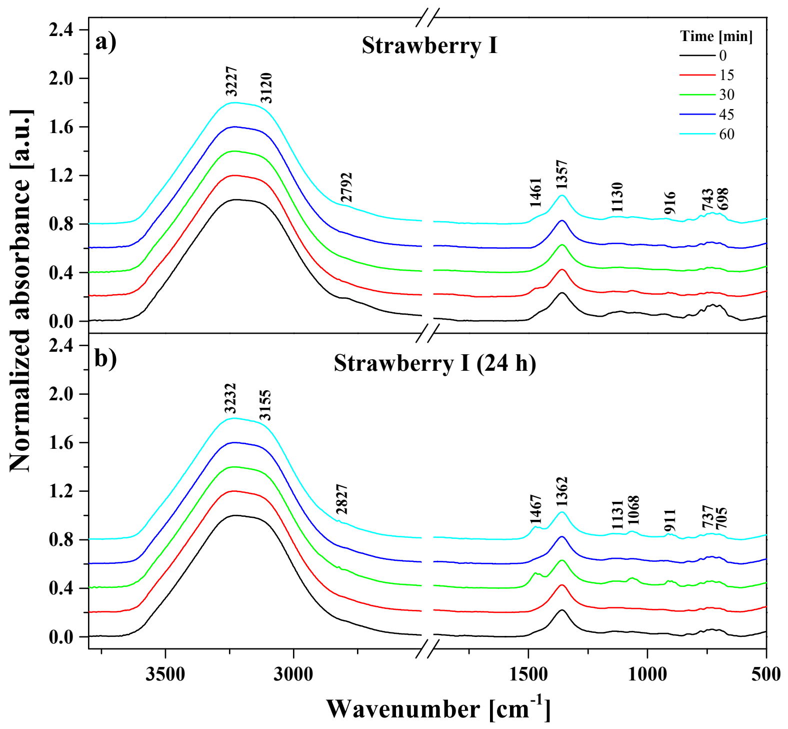

Figure S5. FTIR absorption spectra for the analyzed *Portola* strawberry variety. One part of raspberry fruit was frozen immediately after ozonation (panel a). The second part of the raspberry fruit was stored for 24 h under refrigeration conditions and then also frozen (panel b).

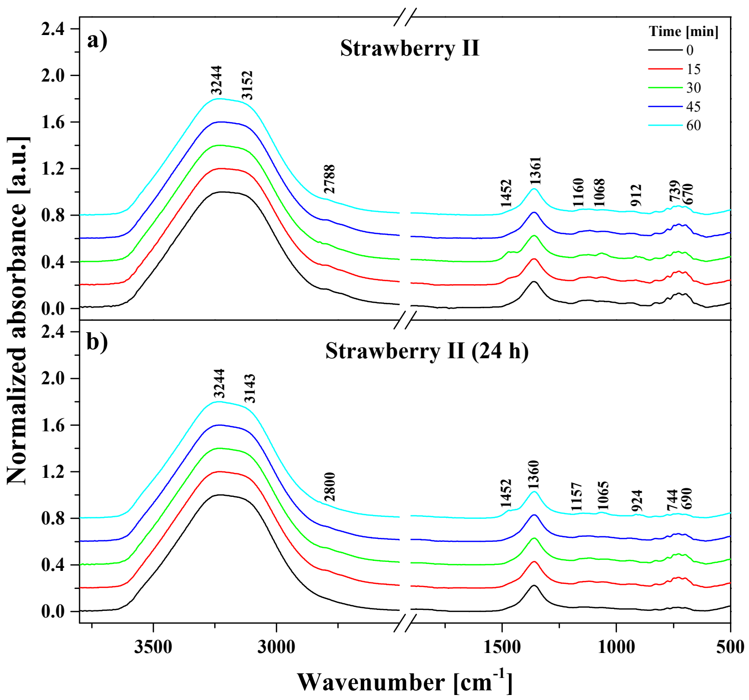

Figure S6. FTIR absorption spectra for the analyzed *Enduro* strawberry variety. One part of raspberry fruit was frozen immediately after ozonation (panel a). The second part of the raspberry fruit was stored for 24 h under refrigeration conditions and then also frozen (panel b).

PCA– FTIR spectra

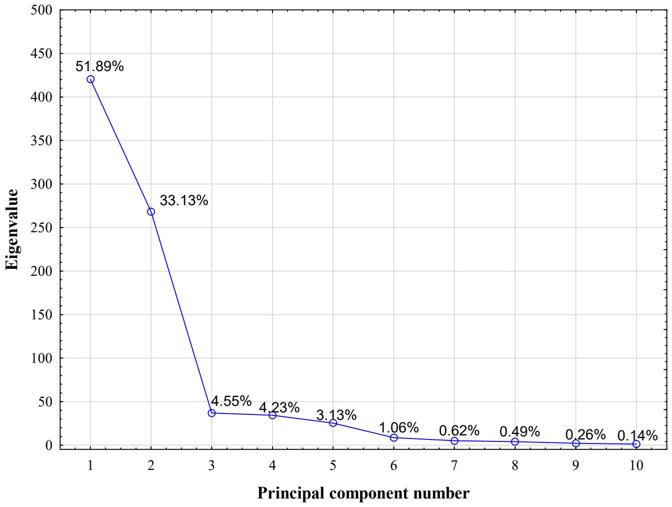

Figure S7. Scree plots (eigenvalues from principal components) for all examined samples of raspberry and strawberry from FTIR spectra (1800–500 cm^{-1} region).

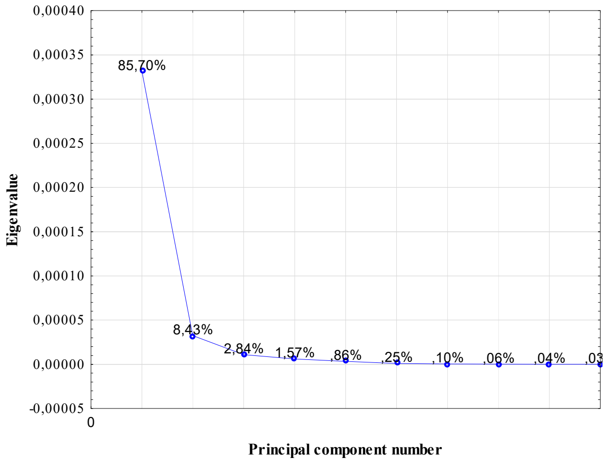

Figure S8. Scree plots (eigenvalues from principal components) for all examined samples of raspberry and strawberry from FTIR spectra (1800–500 cm^{-1} region). The pre-processed (smoothing with 20 windows + 1st derivation) FTIR spectra.
